# Co-administration of calcium and multiple micronutrient supplements for maternal and newborn haemoglobin and iron status: protocol for a randomised non-inferiority trial in Burkina Faso and Pakistan

**DOI:** 10.1136/bmjopen-2026-121658

**Published:** 2026-09-21

**Authors:** Adama Baguiya, Amanda C Palmer, Saleem Jessani, Kerry J Schulze, Melinda K Munos, Monica M Pasqualino, Ethan K Gough, Nicole Cunningham, Fred Van Dyk, Sarah Saleem, Seni Kouanda

**Affiliations:** 1 Institut Africain de Santé Publique, Ouagadougou, Centre Region, Burkina Faso; 2 Institut de Recherche en Sciences de la Santé, Ouagadougou, Centre Region, Burkina Faso; 3 Department of International Health, Johns Hopkins Bloomberg School of Public Health, Baltimore, Maryland, USA; 4 Department of Community Health Sciences, Aga Khan University, Karachi, Pakistan

**Keywords:** Pregnancy, Nutrition, Anaemia

## Abstract

**Introduction:**

The WHO recommends antenatal calcium supplementation (1500–2000 mg/day) to prevent pre-eclampsia in settings with low dietary calcium intake. Implementation is limited partly due to the need for three daily doses, although a lower 500 mg dose may be non-inferior. Current guidelines also recommend separating calcium from iron-containing supplements because concurrent intake may inhibit iron absorption. This individually randomised, controlled, non-inferiority trial will test whether co-administration of calcium and multiple micronutrient supplement (MMS) (CaMMS) is non-inferior to separate administration for haematological and iron status.

**Methods and analysis:**

We will recruit 1600 women at 6<20 weeks’ gestation in Burkina Faso and Pakistan, evaluating non-inferiority independently in each setting, which differ in anaemia aetiology and diets. Participants will be randomly assigned to (a) take United Nations International Multiple Micronutrient Antenatal Preparation Multiple Micronutrient Supplements (30 mg elemental iron) and 500 mg elemental calcium together in the morning or (b) take MMS in the morning and calcium in the evening. Venous blood will be collected at 6<20, 20<24, and 30<34 weeks’ gestation to measure complete blood count, ferritin, soluble transferrin receptor, hepcidin and erythropoietin. The primary outcome is haemoglobin concentration at 30<34 weeks. Co-administration will be considered non-inferior if the lower bound of the 95% CI for the difference in haemoglobin exceeds the non-inferiority margin of –3 g/L. Enrolment commenced in Pakistan on 25 August 2025 and in Burkina Faso on 12 September 2025.

**Ethics and dissemination:**

This protocol was approved by the Johns Hopkins School of Public Health Institutional Review Board, the Comité d’Ethique pour la Recherche en Santé, the Aga Khan University Ethics Review Committee and the Pakistan National Bioethics Committee. Findings will be disseminated through in-country meetings and in the peer-reviewed literature.

**Trial registration number:**

NCT06568315.

**STRENGTHS and LIMITATIONS OF THIS STUDY**

- The calcium and multiple micronutrient supplement (CaMMS) non-inferiority trial testing the impact of co-administering calcium and MMS on haemoglobin and iron status will include sites in both Sub-Saharan Africa and South Asia, enhancing external validity and capturing diverse anaemia aetiologies and background calcium intakes that may influence the nutrient-nutrient interaction.
- The separate administration arm introduces greater complexity that could affect adherence; however, this is mitigated through intensive adherence promotion at monthly dispensing and household visits and further strengthened by unannounced mid-day household visits to assess real-world supplement consumption patterns despite the lack of direct observation.
- Although the trial is not powered for secondary outcomes such as anaemia, iron deficiency and pregnancy outcomes, it is adequately powered for the primary non-inferiority endpoint, which is the main objective informing policy-relevant conclusions.

## Introduction

 Women of reproductive age in low-and-middle-income countries typically consume diets inadequate in many micronutrients.^1^ Micronutrient deficiencies resulting from inadequate intakes are further exacerbated during pregnancy by the increased demands of the developing fetus.^2^ Currently, the WHO recommends daily iron and folic acid (IFA) supplementation with 30 mg to 60 mg of elemental iron and 400 µg of folic acid during pregnancy.^3^ This recommendation is motivated by the burden of anaemia during pregnancy, the specific demand of pregnancy for iron and iron transfer to the fetus. However, they acknowledge that many countries are transitioning from IFA to United Nations International Multiple Micronutrient Antenatal Preparation Multiple Micronutrient Supplements (UNIMMAP MMS or MMS), which includes 15 micronutrients in amounts needed during pregnancy.^4^ Compared with IFA supplementation, MMS improves maternal micronutrient status and increases micronutrient transfer to the fetus.^5–11^ Studies have also revealed a significant reduction of 15% in low birth weight (LBW), 7% in small-for-gestational age (SGA) births, 9% in stillbirth, 19% in early preterm birth (<34 weeks’ gestation) and weaker evidence of a 4% reduction in preterm birth.^12 13^ For countries electing to use MMS in place of IFA, WHO recommends that this action be accompanied by additional research,^4^ for example, on the feasibility, acceptability and cost-effectiveness of MMS versus IFA.

The current formulation of MMS does not contain calcium. Low dietary calcium intake is associated with hypertensive disorders of pregnancy and preeclampsia,^14^ which causes 40 000 maternal deaths annually worldwide.^15^ A 2018 Cochrane meta-analysis of 27 calcium trials^16^ showed that calcium in doses ≥1 g for women with low dietary calcium intake reduced high blood pressure by 35%, preeclampsia by more than half and death or serious morbidity by 20%. The Cochrane review also reported that daily doses of ≥1 g also reduced preterm birth by 24% but had no effect on SGA. In addition to IFA or MMS, WHO recommends that women with low-calcium diets receive 1.5–2.0 g daily in divided doses of 500 mg.^17^ This recommendation has not been widely implemented,^18^ likely due to the cost of supplements and adherence challenges with divided doses. A recently completed non-inferiority (NI) trial carried out in India and Tanzania indicated that a daily 500 mg dose is non-inferior to the higher dose in preventing preeclampsia.^19^ Results from the site in India also suggested that it was non-inferior for the preterm birth outcome.^19^

Given the new evidence that a 500 mg calcium dose is non-inferior to 1500 mg for key public health outcomes,^19^ there is increasing interest in modifying the MMS formulation to include 500 mg elemental calcium. A remaining challenge is that WHO recommends that calcium supplements be taken separately from iron-containing supplements because concurrent ingestion may reduce iron absorption.^17^ However, this supposition was largely based on evidence from studies of iron absorption from single test meals or short-term bioavailability.^20–25^ A 2021 meta-analysis found that, while there was a significant association between the use of calcium supplements and lower serum ferritin concentration, this did not translate into a difference in haemoglobin (Hb) concentration.^26^ It is important to note that most studies had specifically excluded pregnant women and were primarily carried out in high-income country settings, where baseline iron status was likely higher.^26^

A definitive study regarding the impact of concurrent iron and calcium supplementation on the haematological and iron status during pregnancy is an important next step towards the development of a single antenatal supplement that would increase uptake of and adherence to both interventions. The overall goal of the CaMMS trial is to evaluate the impact of co-administration of calcium and MMS, relative to separate administration, on Hb concentration and iron status of pregnant women and their infants.

## Methods and analysis

### Study design

This is an NI trial designed to assess the impact of UNIMMAP MMS and Ca (500 mg) taken concurrently, relative to taking them separately, on haematological and iron status of women and infants. Women will be individually randomised in a 1:1 ratio to take both supplements in the morning (‘together’) or to take the MMS in the morning and the calcium in the evening (‘separate’). Counselling messages and supplement packaging will not enable blinding of participants or study staff with direct participant contact. However, laboratory staff measuring the trial’s outcomes and investigators not directly involved in field work will be blinded. The code will be broken when all outcomes have been assessed. Enrolment commenced in Pakistan on 25 August 2025 and in Burkina Faso on 12 September 2025. Assuming an 8-month recruitment period for Burkina Faso and a 12-month period for Pakistan, we anticipate that field work will be completed by May 2027.

### Study setting

In Burkina Faso, the study will be conducted in Sandbongtenga Province, approximately 100 km northeast of Ouagadougou. The province’s altitude ranges from 350 to 400 m, while rainfall varies between 600 and 750 mm per year. The area is in a highly endemic malaria transmission zone.^27^ Malaria accounts for 57% of hospitalisations and 36% of deaths in health facilities in Burkina Faso. Four out of five cases are due to *Plasmodium falciparum*. Malaria is highly prevalent during the rainy season. Women will be recruited from three antenatal care clinics in Kaya district and two rural medical centres in the neighbouring Boussouma district.

In Pakistan, pregnant women will be recruited from the rural district of Thatta, located 103 km southeast of Karachi in Sindh Province. The district lies within the lower Indus River basin and is predominantly a low-altitude coastal plain, with elevations generally close to sea level. Malaria transmission in Thatta is endemic and follows the seasonal pattern observed across Sindh Province. Notably, Thatta has the highest annual incidence of suspected malaria in Sindh, estimated at approximately 191 cases per 1000 population.^28^ Transmission is characterised by two seasonal peaks: a smaller peak in March, coinciding with the onset of pre-monsoon rains, and a larger peak during the post-monsoon period from August to October, when increased rainfall and standing water provide favourable breeding conditions for Anopheles mosquitoes, the vectors of malaria. Both *Plasmodium vivax* and *P. falciparum* circulate in the area, with *P. vivax* accounting for most reported malaria cases. Trial activities will be carried out in one civil hospital in the district.

### Inclusion & exclusion criteria and recruitment

Inclusion criteria for the trial include: (a) women 6<20 weeks of gestational age, (b) Hb concentration of 70 g/L or higher; (c) willing to attend antenatal care (ANC) in the study clinics; and (d) willing to stop taking IFA and receive the study intervention. Age eligibility will vary by country. In Burkina Faso, we will include married or unmarried pregnant women aged ≥15 years; unmarried pregnant women aged 15–18 years will provide assent, with consent obtained from parents. In Pakistan, we will include married pregnant women ≥18 years. Exclusion criteria include (a) non-viable or extrauterine pregnancy and (b) any contraindications to iron- or calcium-containing supplements. In Burkina Faso, community sensitisation will be carried out in five antenatal care clinic catchment areas in the Kaya Health and Demographic Surveillance Site to promote booking pregnancies in the first trimester. Recruitment will take place when women come for routine antenatal care at one of these five clinics. Women will first see Ministry of Health staff for their exam, which includes a urine pregnancy test and fundal height measurement. Pregnant women with fundal height<22.5 cm will be referred to CaMMS offices adjacent to the antenatal care facility to undergo screening activities. In Pakistan, women will be identified via an ongoing pregnancy surveillance system, which is part of the multi-country Global Network for Women and Children’s Health Research.^29^ Research staff visit all married women of reproductive age to identify women who have missed a menstrual period and offer those women a urine pregnancy test. In addition, Community Informants can provide information on suspected pregnancies to these staff, prompting a household visit. Women with a positive urine pregnancy test who are interested will be accompanied to the Makli Civil Hospital for screening.

At both sites, trained research staff—trial midwives in Burkina Faso or research assistants in Pakistan—will describe the screening process and obtain written consent for screening activities. Following the screening procedures, described below, eligible women will be provided full information on the trial’s purpose, procedures, risks and benefits and will be asked to give their consent to participate using a trial-specific written consent form. In Burkina Faso, consent forms for both the screening activities and trial will be printed in French, the consent process will be conducted in French and a signature will be obtained. If the participant does not understand French or is illiterate, the consent form will be translated orally into the local language (Mooré), which is unwritten, and signed with the presence of a witness who will provide a signature. For unmarried 15–18 year-olds, permission will be obtained from a parent and then assent will be obtained from the adolescent. In Pakistan, written consent will be obtained using forms translated into Sindhi. If consenting women are illiterate, they will be asked to provide a thumb imprint on the consent form in the presence of an independent witness.

### Interventions

All participants will receive two daily supplements: (1) a UNIMMAP MMS, providing 30 mg elemental iron, and (2) a 500 mg elemental calcium supplement as 1250 mg calcium carbonate. In the intervention arm (the ‘together’ arm), women will be instructed to take both tablets together in the morning. In the control arm (the ‘separate’ arm), they will take MMS in the morning and calcium in the evening. Women in both arms will be told to take the supplements starting at enrolment (6<20 weeks’ gestation) until the end of the pregnancy.

**Figure 1:**
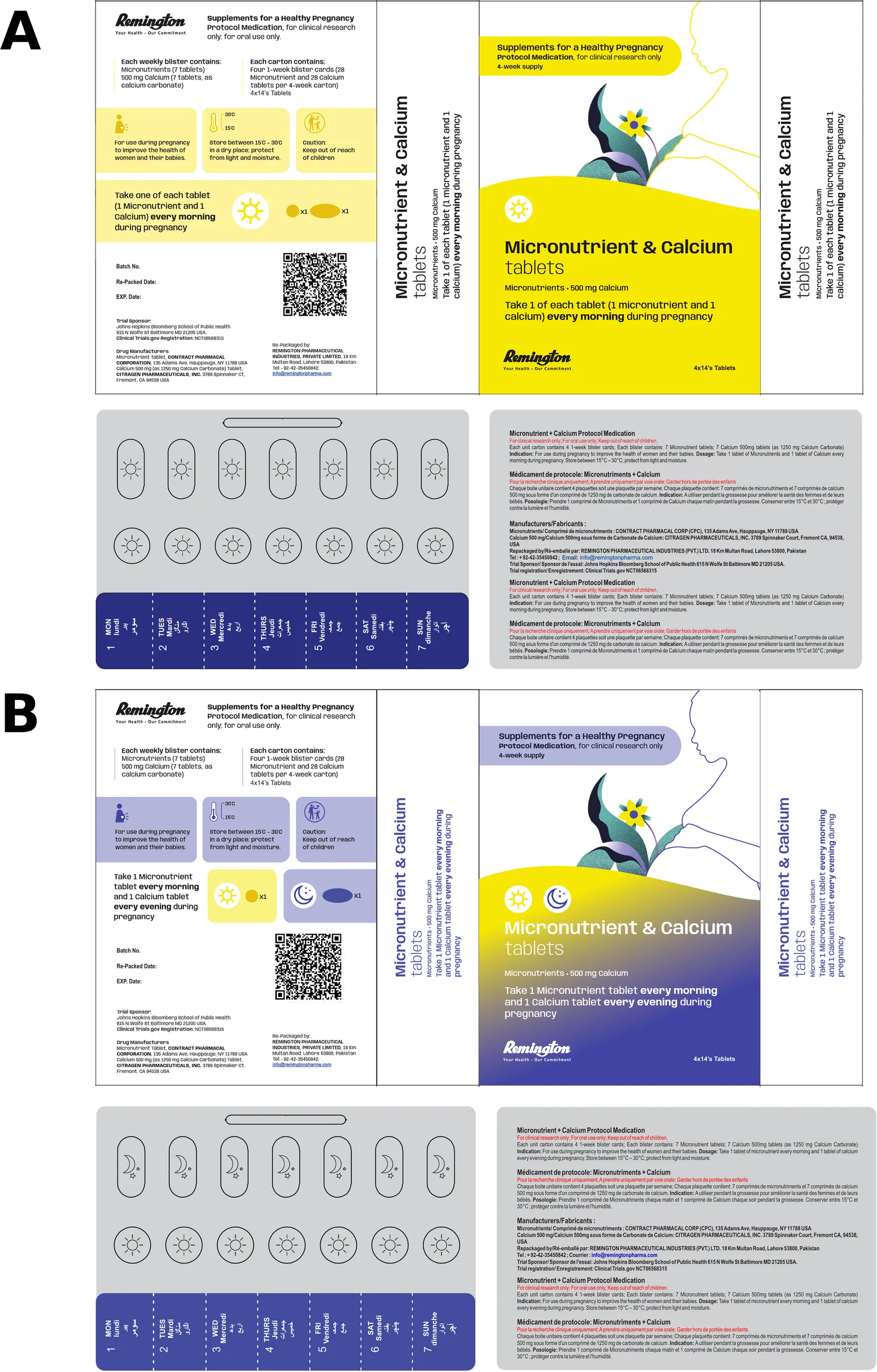
Images of CaMMS trial intervention boxes for (**A**) together arm and (**B**) separate arm. CaMMS, calcium and multiple micronutrient supplement.

UNIMMAP MMS will be sourced from Contract Pharmacal Corporation (Hauppauge, New York, USA). Calcium supplements will be sourced from CitraGen Pharmaceuticals (Fremont, California, USA). Bulk supplements will be co-packaged into arm-specific boxes (figure 1) by Remington Pharmaceuticals (Lahore, Pakistan), with barcoded labels to indicate lot numbers, the repackaging date, and the expiry date of repackaged supplements. Each box will contain four calendar blister packs with a 7-day supply of tablets. Study supplements will be stored in humidity and temperature-controlled pharmacies at each site until delivered to recruitment centres.

As this trial compares two different supplement regimens (ie, taken together vs separately), adherence to the randomised regimen is essential to ensure the validity of the trial results. There is strong evidence that greater medication complexity (ie, multiple drugs, multiple times/day) reduces adherence.^30^ Thus, there is a risk of lower adherence in the arm allocated to separate doses, leading to potential bias. The trial will use a combination of packaging, counselling on key messages and adherence promotion via home visits and adherence partners to promote adherence to the allocated supplement regimen.

Human-centred design work carried out at both sites indicated that attractive packaging and a dedicated place to store supplements could encourage pregnant women to take pills. As calendar blister packaging has been shown to increase adherence compared with bottles,^31–33^ this strategy was also selected. Both boxes and blister cards will be marked to indicate whether women should take both supplements in the morning (marked with a sun icon) or to take the MMS in the morning and calcium in the evening (marked with a sun and moon and stars). The packaging will be explained to the woman at the enrolment visit and reinforced at follow-up visits as necessary.

Counselling cards with key messages, prompts and images were adapted from previous studies and revised based on pretesting of drafted materials and messages in interviews and focus groups. Key messages and adherence materials are available via Open Science Framework (https://doi.org/10.17605/OSF.IO/36W58). Cards will include counselling messages regarding how to take the supplements, adherence strategies and common barriers to adherence and how to address them. Study staff will deliver counselling messages to participants at the time the initial supplements are dispensed and will reinforce these messages at each subsequent visit. The counselling messages include other adherence strategies, such as the use of an ‘adherence partner’; taking the supplements at the same time and/or before/during/after a meal; and keeping the supplements in a visible place, such as the study-provided bag. In addition to centre-based counselling, either community health workers (Burkina Faso) or research staff (Pakistan) will visit women in their households to assess and support adherence, as described below.

### Assignment of interventions

The randomisation schema will be prepared by EKG and FVD using random permuted blocks with variable block size (4, 8 or 12) with a 1:1 allocation to the ‘together’ or ‘separate’ regimen, stratified by site and recruitment clinic. The list will include the sequence, country, health centre, randomly allocated intervention and unique participant identifier. An access-restricted, web-based, centralised allocation system covering both sites will be developed. Once a woman has provided consent for trial enrolment, the research staff will enter his or her valid username and password, the woman’s screening identification number and the enrolment date. They will then press the ‘Request New Allocation’ button and the system will reveal the next allocation to Separate or Together in the randomisation sequence, displaying the woman’s assigned 6-digit alphanumeric identification number.

### Outcomes

Our primary aim is to evaluate the effect of co-administered Ca and MMS on mean Hb concentration at 30<34 weeks of gestation, as measured by complete blood count (CBC).

Our secondary aims are to evaluate the effect of co-administered Ca and MMS on:

- Iron status of pregnant women at 30<34 weeks of gestation (see table 1 for justifications): ferritin, soluble transferrin receptor (sTfR), hepcidin and erythropoietin (EPO) concentrations, as well as sTfR-ferritin index and hepcidin-EPO ratio; ferritin and sTfR will be presented with and without adjustment for inflammation.^34^
- Haematological status of pregnant women at 30<34 weeks of gestation: red blood cell count, mean corpuscular volume, mean corpuscular Hb and mean corpuscular Hb concentration.
- Hb concentration of infants within 72 hours of birth.
- Iron status of infants within 72 hours of birth.

**Table 1:** Secondary iron status outcomes to be used in the CaMMS Trial

Outcome	Justification
Ferritin	Measure of storage iron; as inflammation increases ferritin and can mask true iron deficiency, data will be presented as unadjusted and adjusted using the BRINDA method^34^: Ferritin_adjusted_=ferritin_unadjusted_ – β_1_(CRP_obs_ – CRP_ref_) – β_2_(AGP_obs_ – AGP_ref_) – β_3_(malaria)
Soluble transferrin receptor (sTfR)	Measure of tissue iron demand for erythropoiesis; data will be presented both unadjusted and adjusted for inflammation: sTfR_adjusted_=sTfR_unadjusted_ – β_1_(CRP_obs_ – CRP_ref_) – β_2_(AGP_obs_ – AGP_ref_) – β_3_(malaria)
sTfR:log-ferritin ratio	Expressed to distinguish iron deficiency from apparent deficiency that occurs with inflammation; basis for calculation of total body iron stores
Hepcidin	Regulator of iron absorption; iron sufficiency and inflammation result in increased hepcidin and decreased iron absorption
Erythropoietin (EPO)	Endocrine signal for erythrocyte production
Hepcidin:EPO ratio	More sensitive measure of poor iron status, combining information regarding absorbed and circulating iron with extent of hypoxia

CaMMS, calcium and multiple micronutrient supplement; CRP, C-reactive protein.

Exploratory outcomes, defined as pre-specified outcomes not included in the confirmatory analysis and for which the trial was not powered to detect differences, include anaemia (Hb<110 g/L) at 30<34 weeks and, as an indicator of intervention safety, severe anaemia (Hb<70 g/L) at 20<24 weeks and 30<34 weeks of gestation. Other exploratory outcomes, defined in table 2, include iron deficiency, iron deficient erythropoiesis, iron deficiency anaemia, birth weight, LBW, gestational age at birth, weight-for-gestational age (GA) Z-score, small-for-gestational age, miscarriage and stillbirth.

**Table 2:** Definitions of exploratory outcomes to be assessed in the CaMMS trial

Exploratory outcomes	Definition
Anaemia	Hb<110 g/L at 30<34 weeks of gestation
Intervention safety	Severe anaemia (Hb<70 g/L) at 20<24 weeks or 30<34 weeks
Iron deficiency	Plasma ferritin<15 µg/L at 30<34 weeks of gestation^*^
Iron-deficient erythropoiesis	sTfR concentration>8.3 mg/L at 30<34 weeks of gestation
Iron deficiency anaemia	Concurrent anaemia (Hb<110 g/L) and iron deficiency (plasma ferritin<15 µg/L)^1^ at 30<34 weeks of gestation
Birth weight	Infant weight within 72 hours of birth
Low birth weight	Infant weight<2500 g within 72 hours of birth
Gestational age (GA) at birth	GA at birth in weeks based on screening ultrasound and INTERGROWTH-21st standard
Preterm birth	GA at birth<37 weeks of gestation
Weight-for-gestational age Z-score	Calculated based on weight≤72 hour of birth using the INTERGROWTH-21st standard
Small-for-gestational age	<10th percentile of weight-for-gestational age using INTERGROWTH-21st standard
Miscarriage	Foetal loss <20 gestational weeks
Stillbirth	Foetal loss ≥20 gestational weeks

* An alternative physiologically based ferritin threshold of 25 µg/L will be used in sensitivity analyses based on recent evidence that this higher threshold may better identify individuals at an earlier stage of iron deficiency, when iron stores are insufficient for red blood cell production and other tissue functions.

CaMMS, calcium and multiple micronutrient supplement; Hb, haemoglobin ; sTfR, soluble transferrin receptor.

### Sample size

For this NI trial, we calculated an NI margin using guidelines developed by the US Food and Drug Administration and the European Medicines Agency.^35^ Briefly, the NI margin is based on effect sizes from prior meta-analyses or trials of the active control, with the smallest expected treatment effect (M1) set at the limit of the 95% CI that is closest to the null effect. We found no studies comparing the effects of MMS and calcium taken together versus placebo, and data comparing MMS to a placebo were similarly limited. We therefore used the pooled effect size for Hb concentration at or near term (8.88 g/L (95% CI 6.96 to 10.8)) from 20 trials of iron-containing supplements, compared with the same formulations without iron, no treatment or a placebo.^36^ As the lower limit is clinically large (7.0 g/L), common practice is to preserve 50% of the minimum effect for an NI margin (M2) of −3.5 g/L. However, the absolute value of this effect exceeds 3.0 g/L, which we would consider clinically meaningful based on the difference between the third and fifth normative centiles for maternal Hb at 30<34 weeks’ gestation derived from the INTERGROWTH-21st Project,^37^ that is, the difference between a probable diagnosis of being at high risk versus anaemic. Therefore, we selected a more conservative NI margin of −3.0 g/L.

We assumed a one-sided α=0.025; 90% power; an SD of 17 g/L, based on prior data from both sites and assuming equal group variance across treatment arms^38,39^; and 15% loss to follow-up. Accordingly, a sample of 1600 women per country (3200 women in total) will be required to conclude NI, in each country independently; that is, that the lower limit of the 95% CI around the difference in Hb concentration between the intervention (MMS and Ca taken together) versus control (MMS and Ca taken separately) is greater than −3 g/L.

### Participant timeline

**Figure 2:**
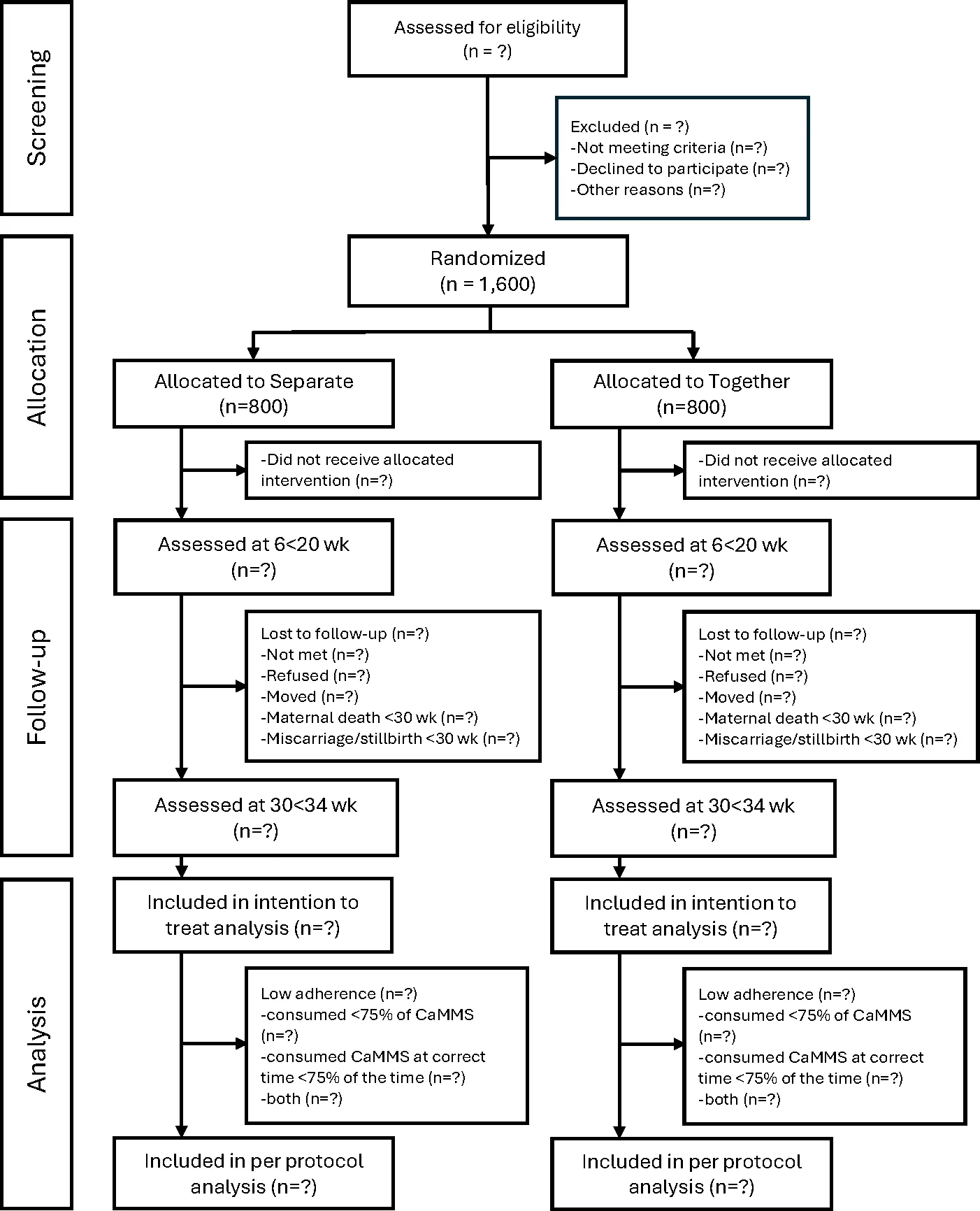
CaMMS trial flow diagram. CaMMS, calcium and multiple micronutrient supplement.

The schedule of enrolment, interventions and assessments is summarised in table 3. Participants will be screened, enrolled and followed monthly in the communities in Pakistan and in health facilities in Burkina Faso. Figure 2 presents a draft flow diagram combining both sites. If notable differences in loss to follow-up or exclusions emerge between sites during analysis, separate diagrams will be provided for each country in the trial manuscripts.

**Table 3:** Schedule of enrolment, interventions and assessments in the CaMMS trial

	Screening	Allocation	Post-allocation					Close-out
TIMEPOINT^*^	*−t_i_* to t_0_	Baseline: 6<20 weeks	Interim ANC/HH visits	Mid-Pregnancy: 20<24 weeks	Interim ANC/HH visits	Late Pregnancy: 30<34 weeks	Interim ANC/HH visits	Birth
		(t_0_)	(t_1_)	(t_2_)	(t_3_)	(t_4_)	(t_5_)	(t_6_)
ENROLLMENT:								
Screening consent	X							
Eligibility screening (Hb testing; ultrasound)	X							
Trial consent	X							
Allocation		X						
INTERVENTION/ COMPARATOR:								
Ca and MMS Together		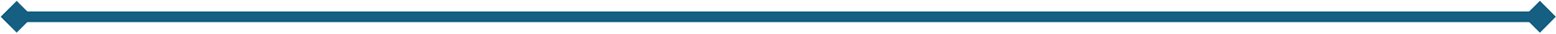						
Ca and MMS Separate		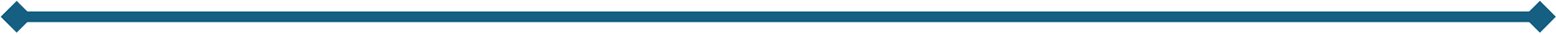						
ASSESSMENTS FOR WOMEN:								
Socio-demographics		X						
Food insecurity		X						
Obstetric history		X						
Dietary intake		X		X		X		
Health card data abstraction^†^		X	X	X	X	X	X	X
Anthropometry		X		X		X		
Blood pressure		X		X		X		X
Venous blood draw		X		X		X		
Point-of-care Hb testing		X		X		X		
Adherence			X	X	X	X	X	X
Morbidity		X	X	X	X	X	X	X
Pregnancy outcome								X
ASSESSMENTS FOR INFANTS:								
Anthropometry								X
Heel prick blood sampling								X
Point-of-care Hb & ferritin testing ^‡^								X

* Interim visits include: (a) routine antenatal care in Burkina Faso or household visits in Pakistan, where data will be collected and supplements dispensed; and (b) HH visits to provide counselling and measure adherence. Timepoints will vary depending on week of enrolment (eg, women enrolling at 6 weeks will have three interim ANC and 4 HH visits; women enrolling at 19 weeks will have no interim ANC and 2–3 HH visits).

† Abstracted data will include anthelminthic medications, sulfadoxine-pyrimethamine (Burkina Faso only), low-dose aspirin, blood transfusion, IV iron, iron-containing supplements.

‡ Point of care ferritin testing will be done for infants only.

ANC, Antenatal care; CaMMS, calcium and multiple micronutrient supplement; Hb, haemoglobin; HH, household; MMS, multiple micronutrient supplement.

### Study visits

#### Screening

Women with a positive urine pregnancy test, fundal height<22.5 cm (in Burkina Faso only) and meeting age criteria will be invited to hear about the trial. Those consenting to screening will be asked about willingness to receive antenatal care at study clinics, discontinue IFA for trial supplements and any contradictions. A capillary blood sample will be taken to measure Hb using a HemoCue Hb 301 device (HemoCue AB, Angelholm, Sweden). Women with Hb<70 g/L will not be enrolled but will be referred for treatment. In Burkina Faso, a malaria rapid diagnostic test will be performed concurrently. All women testing positive will be referred for clinical assessment and treatment according to the national and WHO treatment guidelines, that is, quinine in the first trimester and artemisinin-based combination therapies in the second and third trimesters. Burkina Faso implements the intermittent preventive treatment strategy during pregnancy with sulphadoxine-pyrimethamine (SP). All pregnant women will receive at least three doses of SP one month apart, starting at 16 weeks of gestational age. To ensure full compliance with this strategy, we will supply clinics should they run out of SP stock.

Eligible women will then undergo a transabdominal ultrasound (MindRay DP-10 in Burkina Faso or M6 in Pakistan) to confirm viability and intrauterine pregnancy, assess the number of foetuses, and calculate GA via foetal biometry. Three crown-rump-length (CRL), where CRL≤85 mm, or head circumference measurements will be averaged and GA estimated using INTERGROWTH-21st Standards^40^ or, if<8 weeks, the Verburg reference.^41^ Women with a non-viable or extrauterine pregnancy or with any suspected abnormality will be considered ineligible and referred for care.

#### Baseline

Women meeting all eligibility criteria will be asked to undergo the informed consent procedure for the trial. Consenting women will be randomly allocated as described and research staff will complete a baseline form, capturing data on demographics, socioeconomic status,^42^ household food insecurity,^43^ obstetric history, tobacco use (for Pakistan only) and diet. Dietary data will be collected using a semi-quantitative food frequency questionnaire (FFQ) developed using prior 24-hour recalls collected at each site^44 45^ to capture approximately 95% of iron and calcium intake. Portion sizes were standardised by household utensils, bowl sizes, or reference sizes; weight equivalents and visual aids were developed at each site. Because shared meals are common in Burkina Faso, questions on eating alone versus with others were included; in Pakistan, enumerators were trained to address this. The FFQ was pre-tested at both sites before finalisation.

After administering the questionnaire, women will undergo a physical exam, where staff will measure women’s weight to the nearest 100 g using an ADE M316600 electronic floor scale (ADE Germany GmbH, Hamburg, Germany), height to the nearest 1 mm using an ADE MZ10042 mobile stadiometer (ADE Germany GmbH, Hamburg, Germany), mid-upper arm circumference to the nearest 1 mm, and blood pressure using the Microlife Cradle VSA (Microlife, Widnaw, Switzerland). They will then use antecubital venipuncture to collect a venous blood sample into (a) an untreated or serum-separator blood collection tube for serum-based analyses (ferritin; soluble transferrin receptor; hepcidin; erythropoietin; C-reactive protein (CRP); α_1_-acid glycoprotein (AGP)) and (b) an EDTA-treated evacuated blood collection tube for assays requiring whole blood (CBC; haemoglobinopathies; malaria parasitaemia in Pakistan). While the Hb measured by HemoCue at screening will serve as an initial screening for severe anaemia, results of the CBC will be used for the baseline Hb measurement. CBC results will be reviewed to identify any women with Hb<70 g/L who were not flagged by on-site HemoCue testing. For the baseline visit, these women will be contacted to provide a referral and will be treated as post-randomisation exclusions. Blood samples will be stored in insulated containers with cold packs prior to transit to local laboratories for processing. Finally, women will be given two boxes of study supplements (one 4-week supply and a 4-week buffer) and receive counselling, as described above.

#### ANC follow-up visits

After enrolment, women will be seen by research staff every 4 weeks and encouraged to attend all recommended ANC visits. In Burkina Faso, these visits will coincide with routine antenatal care. In Pakistan, except for the 20<24 and 30<34 week visits, follow-ups will take place at the home. At each visit, adherence will be assessed by asking about supplement use on the prior day, including: whether the woman took each of the supplements, whether they were taken together or separately, at what time each of the supplements was taken, and whether each of the supplements was taken with or without food. Women will also be asked how many days in the past week they took each of the supplements. Finally, we will count and record the number of tablets remaining in the blister packs. Additional supplements will be dispensed so that women always have an 8-week supply. Staff will also collect morbidity information and abstract health card data on anthelminthic medication, sulfadoxine-pyrimethamine (Burkina Faso only), and low-dose aspirin. Providers at both sites will be trained to clearly record any blood transfusions, IV iron, and iron-containing supplements for data abstraction, and women will be asked to keep prescriptions and medication boxes. Information on all locally available iron-containing supplements, including photographs of packaging, was collected to facilitate data capture.

For visits in the windows of 20<24 and 30<34 weeks, women will also undergo a physical exam (including anthropometric measurements and blood pressure) and dietary intake assessment. The exam will include the venous blood draw, as described above. On-site Hb testing will be done using the HemoCue device for the purpose of identifying women with Hb<70 g/L and referring them for care while they are at the clinic. The primary trial outcome and safety outcome will be based on results of the CBC. These results will be reviewed after each visit to identify any cases of severe anaemia that were missed by on-site HemoCue testing. These women will be contacted and referred for care; their outcome data will be retained in the analysis. In Burkina Faso, malaria rapid diagnostic testing will also be done and, if positive, women will be referred for treatment.

#### Adherence visits

Home visits will be conducted by Community Health Workers in Burkina Faso (2–3 times during the first 2 weeks, then monthly) and by research staff in Pakistan (twice in the first 2 weeks, then monthly) to reinforce adherence messages. At each visit, women will be asked about whether they took the MMS tablet on that day and on the previous day, and at what time they took the MMS. The same questions will be asked for the calcium tablet. Staff will also review supplement blister packs to visually confirm adherence to the assigned regimen on the day of the visit. Information on women’s reasons for not taking their supplements as per instructions will be collected to understand barriers. Staff will also review key counselling messages and will provide additional targeted counselling for women who are non-adherent or who have questions about the intervention.

#### Birth visit

A visit will be conducted within 72 hours of birth to collect data on pregnancy outcome, maternal and infant vital status and infant sex. Measurements of maternal blood pressure with the Microlife Cradle VSA and infant weight to the nearest 10 g using the ADE M118600 baby scale (ADE Germany GmbH, Hamburg, Germany) will be done. A capillary blood sample will be taken by heel prick from the infant and tested on site for Hb using the HemoCue Hb 301 and ferritin using a quantitative immunochromographic test run on the Easy Reader+platform (Veda-Lab, Alençon, France) to inform our secondary outcomes (ie, Hb and iron status within 72 hours of birth). Newborns with Hb<110 g/L will be referred for care. Referrals will not be made based on ferritin results; however, these will be provided to the treating clinician in the event of referral for low Hb.

### Patient and public involvement

Participants and members of the study communities were not involved in the design of the trial. However, engagement has occurred through recruitment of local staff to support community sensitisation activities. Plans are in place to involve community members in dissemination of study findings to participants and other local stakeholders.

### Data management

During interviews, most data will be directly entered on tablets into electronic case report forms using REDCap software. Exceptions include names and contact information, which will be maintained only in hardcopy records at both sites. In addition, data collected by Community Health Workers as part of household visits in Burkina Faso will be collected on hardcopy forms and entered into REDCap by research staff. While ultrasound measurements will be entered into electronic CRFs, ultrasound images will be captured and temporarily stored on the ultrasound machines (MindRay DP-10 in Burkina Faso; MindRay M6 in Pakistan), with images transferred on a weekly basis to the local server for back-up. REDCap forms will be configured to include double data entry of unique identifiers and range checks for all continuous variables. All electronic data will be stored in a secure, access-restricted REDCap database. Where data are collected and stored on paper, staff will keep paper private, and forms secured during the workday. At field offices, the paper forms will be stored in locked trunks and cabinets. These local field offices will also be locked, and access will only be permitted to pre-approved study personnel. Data for analysis (largely de-identified) will be hosted on an encrypted cloud server, with daily back-up. The file storage system will be the main data repository for analytic data.

### Biospecimen analysis

Venous blood collected at the early-pregnancy (6<20 weeks), mid-pregnancy (20<24 weeks) and late-pregnancy (30<34 weeks) visits will be stored on ice packs for shipment in EDTA-treated vacutainers and either untreated or serum separator vacutainers. In Burkina Faso, EDTA-treated whole blood will be sent to the Kaya District Hospital for full haematology analysis using a Sysmex MODEL XN-1000 haematology analyser (Sysmex Corporation, Kobe, Japan) and, at the baseline visit, qualitative testing for haemoglobinopathies by agarose electrophoresis on a cellulose acetate strip using a Wincom DY-300 Electrophoresis Machine (Wincom Company, Changsha, China). Ferritin and CRP will be measured using immunoturbidimetric assays on a Biolis 30i Automated Clinical Chemistry Analyzer (Tokyo Boeki Medical System, Tokyo, Japan). An aliquot of serum will be shipped on dry ice to the Micronutrient Laboratory at the Johns Hopkins Bloomberg School of Public Health, where commercial immunoassay kits will be used to measure sTfR (R&D Systems, Minneapolis, Minnesota, USA), hepcidin (DRG International, Springfield, New Jersey, USA), and EPO (R&D Systems, Minneapolis, Minnesota, USA). A commercial radial immunodiffusion assay (Kent Laboratories, Bellingham, Washington, USA) will be used to measure AGP.

In Pakistan, CBCs will be obtained using a Sysmex XN-100 automated haematology analyser (Sysmex Corporation, Kobe, Japan); haemoglobinopathies will be assessed by HPLC performed on a BIORAD Variant II instrument; and malaria parasitaemia will be assessed by microscopy on thick and thin blood smears. Both haemoglobinopathy and malaria testing will be done at baseline only in Pakistan, to inform ancillary analyses on the aetiology of anaemia in this setting. Ferritin, sTfR and CRP will be measured by immunoturbidimetric assay and AGP by immunological agglutination/turbidimetric assay on a Cobas c 311 Automated Analyzer (Roche Diagnostics GmbH, Mannheim, Germany), EPO will be measured using a chemiluminescent assay on the Immulite-2000 platform (Siemens Healthcare Diagnostics, Tarrytown, New York, USA), and hepcidin by a commercial competitive binding immunoassay (DRG Hepcidin 25 bioactive HS ELISA kit, DRG International, Springfield, New York, USA).

Results of ferritin, malaria and haemoglobinopathy testing will be shared with women. Women with haemoglobinopathies will be referred for counselling and medical management and monitoring. As National Pregnancy Care Guidelines in Burkina Faso contraindicate iron supplementation during pregnancy in women with SS or SC haemoglobinopathies, we will apply this exclusion to both sites. They will be deemed ineligible for the trial based on a contraindication and treated as post-randomisation exclusions. Women who are AS or AC carriers will be eligible for the trial and informed of their carrier status. Results of all tests will be kept confidential by the study team, but women will be free to share results with their care providers.

### Statistical methods

The statistical analysis will be structured around three pre-specified estimands, defined in accordance with the ICH E9(R1) Addendum and described in online supplemental table 1. Anticipated intercurrent events and their handling strategies are specified in online supplemental table 2. Briefly, the primary estimand applies a treatment policy strategy across all randomised women (ITT); the secondary estimand applies a per protocol strategy restricted to adherent women; and the sensitivity estimand applies an analysis restricted to women who did not develop severe anaemia prior to outcome ascertainment (mITT). The analyses described below correspond to these three estimands.

For analysis of the primary outcome—Hb measured by haematology analyser at 30<34 weeks of gestation—we will apply all three estimands. For the per protocol analysis, we will use a two-tier approach to characterise adherence. The first tier will quantify the median pill count per monthly follow-up visit. The second tier will quantify the percentage of monthly follow-up visits and household visits where women self-reported taking calcium and MMS at the appropriate time on the previous day, that is, the time prescribed for their assigned study arm. The per protocol dataset will include subjects who consumed a median of ≥75% study supplements per month based on pill count and consumed supplements at the prescribed time on the prior day at ≥75% of visits. The mITT dataset will include subjects who were not diagnosed with severe anaemia (Hb 70 g/L) during follow-up period.

**Figure 3:**
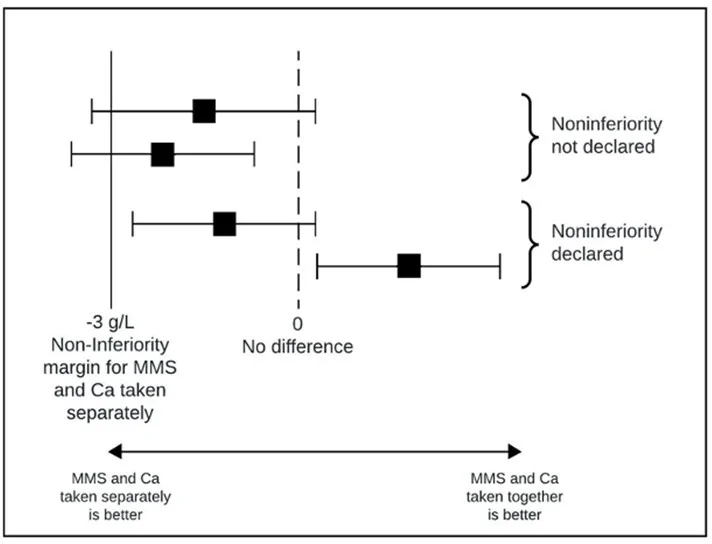
Interpretation of noninferiority results from CIs using the fixed margins method. MMS, multiple micronutrient supplement.

As treatment effects may vary between countries due to differences in the determinants of Hb concentration, we will use a two-stage approach to combine treatment effect estimates across countries. In the first stage, we will estimate country-specific treatment effects and 95% CIs using simple linear regression models with a dummy variable for allocation. We will treat these site-specific analyses as separate hypothesis tests and will not correct for multiple testing across site-specific analysis results. In the second stage, we will pool the country-specific effects. Since between-country heterogeneity in treatment effects cannot be estimated precisely with only two countries, we will present both fixed-effect and random-effects estimates across countries. The latter will be performed using the inverse variance approach with the between-country treatment effect variance estimated by the DerSimonian and Laird method. Country-specific results will be the primary basis for the NI decision; the pooled estimates are secondary summaries to present the range of plausible pooled effects without relying on an imprecise estimate of between-country heterogeneity or assuming a common effect across countries. In addition to the unadjusted analyses, we will conduct sensitivity analyses including the following baseline covariates: parity, body mass index, height and baseline values for Hb, ferritin, CRP and AGP. We will follow the same approach for the ITT, PP and mITT analyses. NI will be determined using the fixed-margin methods (figure 3). That is, if the lower limit of the 95% CI around the difference in Hb concentration between the intervention and control is greater than the NI margin of −3 g/L, the intervention will be deemed non-inferior. We will use both ITT and PP approaches. For NI trials, PP analysis provides a more conservative assessment; however, as differential adherence between arms is possible, PP analysis may introduce bias through differential post-randomisation selection based on adherence. Therefore, while both analyses will be examined for consistency, ITT will be considered primary. We will also explore effect modification based on receipt of any iron treatment, extracted from health cards, and dietary iron and calcium intakes. A *p* value<0.05 for the interaction term will be considered statistically significant.

If <10% of outcomes are missing due to losses to follow-up, specimen handling or testing considerations, or very preterm or extremely preterm birth prior to 30 weeks, we will perform multiple imputation by chained equations of missing outcome data using predictive mean matching to generate 50 complete datasets for ITT analysis.^46^ Multiple imputation has been proposed as a robust strategy to address outcome missingness in NI trials under the assumption that data are missing at random.^47^ Our imputation model will include, at minimum, treatment arm and covariates we have prespecified for inclusion in adjusted models. Multiple imputation will be performed within country to preserve site-specific data structures. Analyses will be repeated on each imputed complete dataset, and results will be pooled using Rubin’s method. We will conduct sensitivity analyses using inverse probability weighting to assess the robustness of conclusions to missingness if ≥10% of outcome data are missing.^48^

The analytic approach for secondary (table 1) and exploratory outcomes (table 2) will use ITT, PP and mITT to determine superiority of Ca and MMS taken together compared with Ca and MMS taken separately. We will use the same two-stage approach to analyse the countries separately and then pool country-specific treatment effects. For ferritin and soluble transferrin receptor, we will present results both unadjusted and adjusted for inflammation (CRP and AGP) using the methods published by Namaste *et al.*^34^ For binary outcomes, we will use log-binomial regression models with a log link and a binomial response distribution to estimate prevalence ratios with 95% CIs. P values will be adjusted for multiple comparisons using the Holm method, and statistical significance will be determined at α=0.05.

A full Statistical Analysis Plan is available at https://doi.org/10.17605/OSF.IO/36W58.

### Monitoring

Internal monitoring will be conducted using a risk-adapted monitoring approach,^49^ leveraging the REDCap database to generate monthly reports for each country and clinic on predefined performance indicators: screening for eligibility, consent and enrolment; frequency of predefined adverse and severe adverse events; timely completion of critical assessments (eg, biospecimen collection); distribution of the trial interventions; adherence; and withdrawals or drop-outs. Monthly reports will be shared with all investigators and, if needed based on performance indicators, additional in-person site visits will be conducted. The trial will also be overseen by a Data Safety and Monitoring Board (DSMB) with expertise in obstetrics, haematology, epidemiology and biostatistics. Members include Dr Shamsa Humayun (Chair; Fatima Jinnah Medical University, Pakistan), Dr Muhammad Hasan (Aga Khan University, Pakistan), Dr Moussa Lingani (Institut de Recherche en Science de la Santé, Burkina Faso), Dr Salam Sawadogo (Université Joseph Ki-Zerbo) and Dr Maria Knoll (Johns Hopkins University, USA). The DSMB will meet on a quarterly basis to review study progress, participant recruitment and retention, site performance, protocol modifications and safety. The DSMB may recommend stopping the trial if there is evidence of a significant difference (p<0.05) in severe anaemia or other SAEs between groups during these quarterly reviews.

## ETHICS AND DISSEMINATION

### Ethical Approvals

This protocol was reviewed by Johns Hopkins School of Public Health Institutional Review Board (IRB00030029), with initial approval granted on 5 January 2025. In Burkina Faso, approval by the Comité d’Ethique pour la Recherche en Santé (2024-08-245) was granted on 13 August 2024. In Pakistan, the protocol received approval from the Aga Khan University Ethics Review Committee (2024-10453-31544) on 14 October 2024 and from the National Bioethics Committee (NBCR-1208) on 10 March 2025. The trial was registered at clinicaltrials.gov as NCT06568315 on 23 August 2024. Clinical trial authorization was secured from the Agence Nationale de Régulation Pharmaceutique in Burkina Faso (N°2025-0002/MS/SG/ANRP) on 3 January 2025 and from the Drug Regulatory Authority (NO.03-65/2025-CT) of Pakistan on 29 April 2025.

### Availability of data and materials

Deidentified data will be made available following publication of the primary outcome manuscript on Zenodo. The full trial protocol, consent materials, statistical analysis plan, data collection materials, and adherence materials have been posted to Open Science Framework (https://doi.org/10.17605/OSF.IO/36W58).

### Dissemination

Participants will be kept informed of the study’s progress and any changes to implementation plans by research staff. Findings will be disseminated to participants and local government officials through community engagement activities. Trial results will be presented to national government officials through dissemination meetings. After results have been shared with communities, local, and national partners, we will present findings at national or international conferences and publish manuscripts both as preprints and in academic journals.

## Supplementary material

10.1136/bmjopen-2026-121658 online supplemental file 1

## Data Availability

No data are available.
