# Supplementary material for: Co-administration of calcium and multiple micronutrient supplements for maternal and newborn haemoglobin and iron status: protocol for a randomised non-inferiority trial in Burkina Faso and Pakistan
Source: BMJ Open. 2026 Sep 21;16(9):e121658. doi: 10.1136/bmjopen-2026-121658 (PMC13599970; doi:10.1136/bmjopen-2026-121658)
Supplement: Supplementary file 1 [file bmjopen-16-9-s001.pdf]

Supplemental Table 1: Estimands framework for the CaMMS Trial

	**Estimand 1: Primary Estimand**	**Estimand 2: Secondary Estimand**	**Estimand 3: Sensitivity Estimand**
**Population**	All randomized women (intention-to-treat population)	Women who consumed ≥75% of study supplements per month by pill count AND took supplements at the prescribed time at ≥75% of follow-up visits (per-protocol population)	All randomized women who did not develop severe anemia (Hb <70 g/L) at any follow-up visit prior to ascertainment of the primary outcome (modified intention-to-treat population)
**Variable**[1]	Hemoglobin concentration (g/L) at 30<34 weeks of gestation	Hemoglobin concentration (g/L) at 30<34 weeks of gestation	Hemoglobin concentration (g/L) at 30<34 weeks of gestation
**Summary measure**	Difference in mean Hb concentration (co-administration minus separate administration), with 95% confidence interval	Difference in mean Hb concentration (co-administration minus separate administration), with 95% confidence interval	Difference in mean Hb concentration (co-administration minus separate administration), with 95% confidence interval
**Intercurrent event handling**	Treatment policy strategy: all outcomes are analyzed regardless of adherence to the study regimen, receipt of non-study iron, development of severe anemia, or other intercurrent events occurring after randomization.	Principal stratum strategy for non-adherence: the treatment effect is estimated within the stratum of women who adhered to the prescribed regimen. All other intercurrent events are handled by the treatment policy strategy.	Principal stratum strategy for severe anemia: the treatment effect is estimated within the stratum of women who did not develop severe anemia during follow-up, and who therefore were unlikely to receive additional therapeutic iron from healthcare providers that would affect the primary outcome independently of the intervention. All other intercurrent events are handled by the treatment policy strategy.
**Clinical question**	What is the effect of assignment to co-administration versus separate administration of MMS and calcium on maternal Hb at 30<34 weeks, as it would occur in practice?	What is the effect of actually taking MMS and calcium co-administered versus separately on maternal Hb at 30<34 weeks, among women who adhere to the prescribed regimen?	Is the primary non-inferiority result robust to the exclusion of women who received additional iron treatment due to severe anemia prior to outcome ascertainment?
**Aligned analysis**	Intention-to-treat (ITT)	Per-protocol (PP)	Modified intention-to-treat (mITT)

[1] Country-specific difference in mean Hb concentration (co-administration minus separate administration), estimated using simple linear regression within each country, with country-specific effects combined as a pooled average treatment effect using a random-effects inverse variance approach (DerSimonian-Laird). Country-specific results are the primary basis for the non-inferiority decision; the pooled estimate is a secondary summary.

Supplemental Table 2: Anticipated intercurrent events in the CaMMS Trial

Intercurrent event	Strategy	Rationale
Non-adherence to study supplements	Treatment policy (ITT); principal stratum (PP)	ITT estimates real-world effectiveness regardless of adherence. PP estimates efficacy among women who adhered to the prescribed regimen.
Receipt of non-study iron supplements or iron therapy	Treatment policy (primary); effect modifier (secondary)	Non-study iron affects Hb independently of treatment arm. Assessed as a potential effect modifier in secondary analyses using an interaction term.
Severe anemia (Hb <70 g/L) requiring referral and treatment	Treatment policy (ITT); principal stratum (mITT)	mITT addresses potential contamination of the primary outcome Hb by therapeutic iron administered following severe anemia diagnosis prior to outcome ascertainment.
Blood transfusion	Treatment policy	Treated as part of the real-world clinical treatment pathway. Occurrence tabulated by arm.
Malaria	Treatment policy (primary); effect modifier (secondary)	Malaria affects Hb independently of intervention. Assessed as a potential effect modifier in secondary analyses.
Pregnancy loss before outcome window (miscarriage or stillbirth)	Missing outcome; multiple imputation under MAR	Primary outcome is unobservable. Treated as missing and handled via multiple imputation by chained equations under a missing-at-random assumption, with inverse probability weighting as a sensitivity analysis.
Very preterm birth (<30 weeks) prior to outcome window	Missing outcome; multiple imputation under MAR	Primary outcome cannot be ascertained within the pre-specified 30<34-week window. Treated as missing and handled via multiple imputation.
