# Supplementary material for: Co-administration of calcium and multiple micronutrient supplements for maternal and newborn haemoglobin and iron status: protocol for a randomised non-inferiority trial in Burkina Faso and Pakistan
Source: BMJ Open. 2026 Sep 21;16(9):e121658. doi: 10.1136/bmjopen-2026-121658 (PMC13599970; doi:10.1136/bmjopen-2026-121658)
Supplement: Supplementary file 2 [file bmjopen-2026-121658.reviewer_comments.pdf]

PEER REVIEW HISTORY

BMJ Open publishes all reviews undertaken for accepted manuscripts. Reviewers are asked to complete a checklist review form and are provided with free text boxes to elaborate on their assessment. These free text comments are reproduced below.

ARTICLE DETAILS

Title (Provisional)

Co-administration of calcium and multiple micronutrient supplements for maternal and newborn haemoglobin and iron status: protocol for a randomized non-inferiority trial in Burkina Faso and Pakistan

Authors

Baguiya, Adama; Palmer, Amanda C.; Jessani, Saleem; Schulze, Kerry J.; Munos, Melinda K.; Pasqualino, Monica M.; Gough, Ethan K.; Cunningham, Nicole; Van Dyk, Fred; Saleem, Sarah; Kouanda, Seni

VERSION 1 - REVIEW

**Reviewer**	**1**
**Name**	**Suchdev, Parminder**
**Affiliation**	**Emory University, School of Public Health**
**Date**	**28-May-2026**
**COI**	**None**

This is a timely and policy-relevant protocol as many countries are scaling up antenatal MMS, while calcium supplementation for prevention of pre-eclampsia remains a separate and parallel antenatal nutrition strategy. A trial directly evaluating whether calcium and MMS can be co-administered without compromising maternal hemoglobin or iron status is highly relevant to implementation and future MMS formulation. I have several major comments related to the trial design, outcome definitions, and reporting plan.

1. Inflammation assessment should be more clearly integrated into interpretation of iron status: The protocol notes measurement of CRP and AGP in the biospecimen analysis section and refers to BRINDA adjustment in Table 1, but this should be made more explicit in the outcomes and statistical analysis sections. Inflammation adjustments will be particularly important given the two trial settings, where malaria, infection, and other inflammatory exposures may differ and may affect apparent treatment effects on iron status. Updated guidance on adjusting iron biomarkers in pregnancy have been published by the BRINDA group: https://pmc.ncbi.nlm.nih.gov/articles/PMC9194318/

2. Ferritin cutoffs for iron deficiency

The protocol defines iron deficiency as plasma ferritin <12 at 30–34 wks gestation. This cutoff may underestimate physiologically meaningful iron deficiency given newer evidence suggesting that higher ferritin thresholds may better identify iron-deficient erythropoiesis (both global and NHANES-based analyses: https://pubmed.ncbi.nlm.nih.gov/40288394, https://pubmed.ncbi.nlm.nih.gov/34329578/). At minimum, the authors should consider sensitivity analyses using alternative ferritin thresholds, for example <15, <25, or <30 μg/L, particularly because iron status is a central secondary/tertiary outcome.

3. Definition of anemia by trimester

The protocol defines anemia as Hb <110 g/L at 30–34 weeks. Since Hb changes physiologically across pregnancy because of hemodilution, the authors should clarify whether trimester- or gestational-age-specific anemia cutoffs will be considered. A single cutoff may not reflect expected mid-pregnancy nadir and later gestational changes. The authors should explain whether they will use the new WHO trimester-specific thresholds or other gestational-age-specific references in sensitivity analyses.

4. Accounting for plasma volume expansion

The primary outcome is Hb concentration at 30–34 weeks. As noted earlier, Hb in pregnancy is influenced not only by iron status, but also by plasma volume expansion. If calcium/MMS co-administration has no biological effect on erythropoiesis but groups differ in gestational age at measurement, nutritional status, inflammation, blood pressure/preeclampsia risk, or plasma volume expansion, Hb differences could be difficult to interpret. The authors should discuss how physiologic hemodilution will be handled analytically. For example, models could adjust for gestational age at blood draw, and anthropometry, blood pressure, EPO, hepcidin, or other markers could help interpret whether differences in Hb reflect iron-related effects versus plasma volume effects.

5. Clarify point-of-care Hb and ferritin testing

Table 3 lists point-of-care Hb and ferritin testing. The methods describe HemoCue testing for screening and safety referral, and newborn ferritin testing using Easy Reader+ within 72 hours of birth. The authors should clarify whether point-of-care ferritin is performed only for newborns or also for pregnant women, and whether these values are used for clinical referral, trial outcomes, or both. For Hb, the protocol should clearly distinguish between point-of-care HemoCue Hb used for screening/safety and venous CBC used for the primary outcome. This distinction is important because point-of-care and venous CBC values may not be interchangeable.

6. Statistical methods.

It would be helpful to clarify whether the primary non-inferiority analysis will be based on each country separately, the pooled estimate, or both. If the trial is intended to inform policy across settings, the decision rule for discordant results between Burkina Faso and Pakistan should be specified a priori. Second, the choice to use simple linear regression with Hb as the outcome and allocation as a predictor seems under-specified. Suggest adjusting for variables such as baseline Hb, GA, and other prespecified prognostic factors. Third, I am unclear about missing data plan. The text notes multiple imputation will be performed “If <10% of outcomes are missing due to losses to follow-up.” Should this be >10%? And will imputation be done by treatment arm and country? Finally, the statistical section should clarify whether analyses will account for repeated measures over pregnancy.

**Reviewer**	**2**
**Name**	**Verhoef, Hans**
**Affiliation**	**Wageningen UR, Division of Human Nutrition and Health**
**Date**	**09-Jun-2026**
**COI**	**None**

bmjopen-2026-121658 Co-administration of calcium and multiple micronutrient supplements for maternal and newborn haemoglobin and iron status: protocol for a randomized non-inferiority trial in Burkina Faso and Pakistan

1. Abstract: reduce introduction substantially. State the objective under a separate heading or include with the introduction.

2. In accordance with ICH E9(R1) Addendum and SPIRIT 2013 guidance, the protocol would be strengthened by explicitly defining the study estimand(s), including the treatment effect of interest and the corresponding analysis population and summary measure. In addition, the authors should identify anticipated intercurrent events (e.g., treatment discontinuation, non-adherence, use of non-study supplements or iron therapy, blood transfusion, severe anaemia/transfusions, malaria, pregnancy loss, and maternal or infant death) and specify how these will be handled within the estimand framework. This would improve clarity regarding the clinical question being addressed and the interpretation of the trial results. Also describe how ITT, PP and mITT analyses (L. 448-454) will be aligned with these estimands.

3. L. 175-181: Add descriptors of the altitude of the study areas (important to interpret haemoglobin concentrations). In the description of the study area in Burkina Faso, add that it is located in a highly endemic, seasonal hyperendemic malaria transmission zone (indicate transmission season). Also describe malaria transmission intensity in the Pakistani study location.

4. Describe the recruitment procedures (where will recruitment take place, what is the source population, how are participants identified, how they were approached, how and where will they be screened for eligibility, how will consent be obtained, etc.?)

5. How will adherence be assessed and analysed?

6. Have the authors considered excluding women with multiple pregnancies? Compared to women with singleton pregnancies, they have higher iron and calcium requirements, a greater risk of iron deficiency and anaemia, higher risk of preterm birth and other complications, and different Hb and iron trajectories during pregnancy.

7. L. 258: ‘their’ should read ‘her’.

8. L. 274: describe what you mean by ‘tertiary outcomes’ (not universally defined in CONSORT or ICH E9), and how they differ from secondary outcomes.

9. L. 274-275: as an indicator of intervention safety, why limit to severe anaemia at 20<24 weeks of [sic] 30<34 weeks of gestation only, and not any incident case of severe anaemia (and/or blood transfusion) during follow-up?

10. L. 298: provide reference for the SD value for haemoglobin concentration (or justify otherwise). Does this SD refer to each group (assuming equal group variance), or to the difference in haemoglobin concentration between groups? If it refers to SD within each group, a value of 17 g/L seems high considering that they will be analysed by Sysmex haematology analyser (L. 432).

11. L. 299: provide clarify if, as I suspect, the sample size of 1,600 is based on the total number (i.e. both intervention groups) within each country (hence, 3,200 in the two countries combined).

12. L. 317-318, 368-373: at baseline and during visits in the windows of 20<24 and 30<34 weeks, Burkinabe women will be tested for malaria using rapid dipstick test. Will positive cases be treated using artemisinin-based combination therapy (2nd and 3rd trimester) or quinine-clindamicin (1st trimester), in accordance with WHO guidelines? Will women also receive intermittent preventive treatment with sulphadoxine-pyrimethamine through their pregnancy?

13. L. 438: Pakistani women will also be tested for malaria, but it is not clear from the description of the ANC visits at what time points.

14. L. 390-391: indicate whether the ferritin assay is qualitative, quantitative or semi-quantitative.

15. L. 424-425, 437-438: please describe ethical issues around the testing for haemoglobinopathies (e.g., sickle cell status) and how they will be dealt with. In particular, will participants always be informed about the results?

16. L. 448-454 (see also my first comment): when will you consider non-inferiority? PP often considered primary or at least co-primary for inference; non-inferiority is typically concluded only if PP analysis shows non-inferiority and ITT analysis is consistent (does not contradict PP).

17. Statistical analysis: The protocol proposes pooling treatment effects across the two countries. This is acceptable if an overall treatment effect is the target estimand, but this should be explicitly stated and justified. The authors should clarify whether country is considered a potential effect modifier and specify how between-country heterogeneity will be assessed (e.g., inclusion of a treatment-by-country interaction term and reporting of country-specific estimates). With only two countries, random-effects pooling should be used cautiously and, if applied, treated as exploratory or sensitivity analysis rather than primary inference. It would seem to make more sense to handle country as a fixed effect.

18. L. 486-88: A log-binomial regression model with a log link estimates risk ratios, not risk differences. I also presume that ‘risk’, at least in some cases (e.g., anaemia, iron deficiency), in fact refers to ‘prevalence’?

19. References 12, 13: issue/page numbers are missing.

VERSION 1 - AUTHOR RESPONSE

Responses to Reviewer 1:

1. Inflammation assessment should be more clearly integrated into interpretation of iron status: The protocol notes measurement of CRP and AGP in the biospecimen analysis section and refers to BRINDA adjustment in Table 1, but this should be made more explicit in the outcomes and statistical analysis sections. Inflammation adjustments will be particularly important given the two trial settings, where malaria, infection, and other inflammatory exposures may differ and may affect apparent treatment effects on iron status. Updated guidance on adjusting iron biomarkers in pregnancy have been published by the BRINDA group: https://pmc.ncbi.nlm.nih.gov/articles/PMC9194318/

   Response: *We agree that the BRINDA adjustments will be important in interpreting the iron status biomarkers. We have added specific statements about biomarker adjustment to the "Outcomes" and "Statistical methods" sections (Lines 305 to 307 and 601 to 603, respectively). We have also added the BRINDA equations to Table 1. As the link above is for an abstract, we have retained the reference to the 2017 methods manuscript by Namaste et al published in AJCN.*

2. Ferritin cutoffs for iron deficiency: The protocol defines iron deficiency as plasma ferritin <12 at 30–34 wks gestation. This cutoff may underestimate physiologically meaningful iron deficiency given newer evidence suggesting that higher ferritin thresholds may better identify iron-deficient erythropoiesis (both global and NHANES-based analyses: https://pubmed.ncbi.nlm.nih.gov/40288394, https://pubmed.ncbi.nlm.nih.gov/34329578/). At minimum, the authors should consider sensitivity analyses using alternative ferritin thresholds, for example <15, <25, or <30 μg/L, particularly because iron status is a central secondary/tertiary outcome.

   Response: *We thank the reviewer for raising this issue. We agree that this would be a valuable analysis. We have retained the WHO threshold in the definition for our exploratory outcome but have indicated in Table 2 that the physiologically based threshold of 25 μg/L will be used in sensitivity analyses.*

3. Definition of anemia by trimester: The protocol defines anemia as Hb <110 g/L at 30–34 weeks. Since Hb changes physiologically across pregnancy because of hemodilution, the authors should clarify whether trimester- or gestational-age-specific anemia cutoffs will be considered. A single cutoff may not reflect expected mid-pregnancy nadir and later gestational changes. The authors should explain whether they will use the new WHO trimester-specific thresholds or other gestational-age-specific references in sensitivity analyses.

   Response: *We agree that the use of trimester-specific cut-offs would be important to consider for the 20<24-week timepoint. However, our categorical exploratory outcome is anaemia at 30<34 weeks, for which we will use the WHO's 3rd trimester-cutoff of <110 g/L.*

At 20<24 weeks, we are considering only severe anemia, primarily for the purpose of monitoring safety. The cut-offs for severe anaemia are unchanged between the 2nd and 3rd trimesters under WHO's 2024 revised thresholds.

4. Accounting for plasma volume expansion: The primary outcome is Hb concentration at 30–34 weeks. As noted earlier, Hb in pregnancy is influenced not only by iron status, but also by plasma volume expansion. If calcium/MMS co-administration has no biological effect on erythropoiesis but groups differ in gestational age at measurement, nutritional status, inflammation, blood pressure/preeclampsia risk, or plasma volume expansion, Hb differences could be difficult to interpret. The authors should discuss how physiologic hemodilution will be handled analytically. For example, models could adjust for gestational age at blood draw, and anthropometry, blood pressure, EPO, hepcidin, or other markers could help interpret whether differences in Hb reflect iron-related effects versus plasma volume effects.

   Response: *We thank the reviewer for raising this point. We agree that hemodilution is important for interpreting the primary Hb outcome. We have revised the "Statistical methods" section (Lines 568 to 570) to pre-specify a sensitivity analysis adjusting for parity, baseline nutritional status, and gestational age at blood draw, which are values that would be considered independent of the intervention. We will also update our Statistical Analysis Plan to indicate that these were selected a priori for their known prognostic relevance to hemodilution, rather than selected post hoc based on any observed between-arm imbalance.*

   EPO, hepcidin, blood pressure, and inflammation markers measured at the time of the primary outcome (30<34 weeks) are intercurrent measurements, i.e., they were collected after randomization and potentially influenced by calcium/MMS supplementation itself. FDA guidance on covariate adjustment in randomized trials cautions against adjusting for such post-baseline variables, since doing so risks conditioning on a potential mediator of the treatment effect rather than a true confounder, which would alter the estimate and could bias the non-inferiority comparison. For this reason, we would not include them as covariates in the confirmatory model. EPO and hepcidin will instead be examined in secondary analyses (Table 2) to help distinguish iron-related/erythropoietic effects from plasma-volume effects mechanistically, without using them to adjust the confirmatory non-inferiority comparison.

5. Clarify point-of-care Hb and ferritin testing: Table 3 lists point-of-care Hb and ferritin testing. The methods describe HemoCue testing for screening and safety referral, and newborn ferritin testing using Easy Reader+ within 72 hours of birth. The authors should clarify whether point-of-care ferritin is performed only for newborns or also for pregnant women, and whether these values are used for clinical referral, trial outcomes,

or both. For Hb, the protocol should clearly distinguish between point-of-care HemoCue Hb used for screening/safety and venous CBC used for the primary outcome. This distinction is important because point-of-care and venous CBC values may not be interchangeable.

Response: *For infants, the point-of-care testing of hemoglobin and ferritin will be used for our secondary trial outcomes, as venous blood collection is not standard practice for neonatal research in these settings. The HemoCue results will also be used to guide any referrals. We will not refer infants based on their ferritin result. However, ferritin results will be provided to the treating clinician in the event of a referral for low Hb. We have clarified this in the "Birth Visit" section (Lines 463 to 465).*

We will not use the point-of-care device to measure ferritin in pregnant women, as this analyte will be measured in the serum samples. We have expanded the "Baseline" section (Lines 400 to 401 and 434 to 441, respectively, as well as Table 3) to clarify that HemoCue results will only be used for eligibility determination and to guide referrals, as well as to indicate that the iron analytes will be measured in serum. As we receive the CBC results, we will review these to identify cases of severe anemia that may have been missed by the HemoCue. For the screening/baseline visit, these women will be treated as post-randomization exclusions. For the follow-up visits, women will be contacted and referred for care. The CBC data will be used to inform both the primary and the safety outcomes.

6. Statistical methods: It would be helpful to clarify whether the primary non-inferiority analysis will be based on each country separately, the pooled estimate, or both. If the trial is intended to inform policy across settings, the decision rule for discordant results between Burkina Faso and Pakistan should be specified a priori. Second, the choice to use simple linear regression with Hb as the outcome and allocation as a predictor seems under-specified. Suggest adjusting for variables such as baseline Hb, GA, and other prespecified prognostic factors. Third, I am unclear about missing data plan. The text notes multiple imputation will be performed "If <10% of outcomes are missing due to losses to follow-up." Should this be >10%? And will imputation be done by treatment arm and country? Finally, the statistical section should clarify whether analyses will account for repeated measures over pregnancy.

Response: *We have revised the "Statistical methods" section to indicate that the country-specific effects will be our primary analyses (Lines 564 to 568). As we have specified in our statistical analysis plan that we will present a pooled analysis, we will report these even if the country-specific results are discordant. However, in our discussion we would stress that pooled results should be interpreted with caution. As noted above and reflected in our*

revised manuscript, we will also present an adjusted analysis, including key baseline variables and gestational age at the time of the blood draw.

For missing outcome data, there are no universally accepted thresholds defining when analyses should be considered exploratory based on the extent of outcome missingness in RCTs. We therefore pre-specify a conservative threshold of <10% missing outcome data. If ≥10% of outcome data are missing, we will conduct complete-case analyses as the primary analysis and perform sensitivity analyses using inverse probability weighting to assess robustness to missingness. Multiple imputation will be performed within country to preserve site-specific data structures. Imputation models will include, at minimum, treatment arm and all covariates prespecified for inclusion in adjusted analyses.

The primary outcome is hemoglobin at 30<34 weeks. We will not account for repeated measures over pregnancy in the main trial manuscript. In our statistical analysis plan, we have described secondary longitudinal data analyses.

Responses to Reviewer 2:

1. Abstract: reduce introduction substantially. State the objective under a separate heading or include with the introduction.

   Response: *We have substantially reduced the introduction and incorporated the objective into it, in line with the journal's abstract structure.*

2. In accordance with ICH E9(R1) Addendum and SPIRIT 2013 guidance, the protocol would be strengthened by explicitly defining the study estimand(s), including the treatment effect of interest and the corresponding analysis population and summary measure. In addition, the authors should identify anticipated intercurrent events (e.g., treatment discontinuation, non-adherence, use of non-study supplements or iron therapy, blood transfusion, severe anaemia/transfusions, malaria, pregnancy loss, and maternal or infant death) and specify how these will be handled within the estimand framework. This would improve clarity regarding the clinical question being addressed and the interpretation of the trial results. Also describe how ITT, PP and mITT analyses (L. 448-454) will be aligned with these estimands.

   Response: *We thank the reviewer for this suggestion. In accordance with ICH E9(R1) and SPIRIT 2013 guidance, we have added two supplemental tables to the manuscript. Supplemental Table 1 defines the study estimands, including the treatment effect of interest, analysis population, summary measure, and the clinical question addressed by each estimand. Supplemental Table 2 specifies anticipated intercurrent events —*

including treatment non-adherence, receipt of non-study iron supplements, severe anaemia requiring treatment, blood transfusion, malaria, pregnancy loss, very preterm birth prior to the outcome window, and maternal death — and the strategy used to handle each within the estimand framework.

3. L. 175-181: Add descriptors of the altitude of the study areas (important to interpret haemoglobin concentrations). In the description of the study area in Burkina Faso, add that it is located in a highly endemic, seasonal hyperendemic malaria transmission zone (indicate transmission season). Also describe malaria transmission intensity in the Pakistani study location.

   Response: *We have provided further information on the altitude and malaria transmission in the "Study setting" section of the revised manuscript (Lines 180 to 186 and 189 to 200).*

4. Describe the recruitment procedures (where will recruitment take place, what is the source population, how are participants identified, how they were approached, how and where will they be screened for eligibility, how will consent be obtained, etc.?)

   Response: *We have expanded the "Inclusion & exclusion criteria and recruitment" section of the manuscript (Lines 210 to 240) to provide additional details on the recruitment and consent procedures.*

5. How will adherence be assessed and analysed?

   Response: *We have expanded the description of adherence assessment in the "ANC Follow-up Visit" and "Adherence Visit" sections (Lines 416 to 423 and Lines 445 to 450, respectively) to indicate the specific questions and recall periods that will be used to assess adherence. We have also expanded the analysis section (Lines 541 to 546) as follows: "For the per protocol analysis, we will use a two-tier approach to characterize adherence. The first tier will quantify the median pill count per monthly follow-up visit. The second tier will quantify the percentage of monthly follow-up visits and household visits where women self-reported taking calcium and MMS at the appropriate time on the previous day, i.e., the time prescribed for their assigned study arm."*

6. Have the authors considered excluding women with multiple pregnancies? Compared to women with singleton pregnancies, they have higher iron and calcium requirements, a greater risk of iron deficiency and anaemia, higher risk of preterm birth and other complications, and different Hb and iron trajectories during pregnancy.

Response: *We thank the reviewer for this suggestion. Based on population surveillance data at both study sites, rates of multiple pregnancies were expected to be low. We did not therefore consider this as a specific exclusion criterion. However, we have added multiple pregnancy as a variable to be reported at baseline, which will allow us to account for this in sensitivity analyses if necessary.*

7. L. 258: ‘their’ should read ‘her’.

   Response: *We have replaced this with “his or her” for clarity, in reference to our study personnel.*

8. L. 274: describe what you mean by ‘tertiary outcomes’ (not universally defined in CONSORT or ICH E9), and how they differ from secondary outcomes.

   Response: *We agree that "tertiary outcomes" is not a universally defined term in CONSORT or ICH E9. We have revised the manuscript (Line 314) and Table 2 to replace this terminology with "exploratory outcomes," defined as pre-specified outcomes that will not be included in the confirmatory analysis and for which the trial was not powered to detect differences.*

9. L. 274-275: as an indicator of intervention safety, why limit to severe anaemia at 20<24 weeks of [sic] 30<34 weeks of gestation only, and not any incident case of severe anaemia (and/or blood transfusion) during follow-up?

   Response: *We limited our safety outcomes to severe anaemia as measured by our research staff using the CBC, which is only being done at 20<24 weeks and 30<34 weeks. In our formative work, we determined that hemoglobin is not consistently measured or documented by healthcare providers at non-study ANC visits. There were also differences in these practices between the two sites. While we are abstracting these data from health records, we are unsure about the quality of these data and, in discussions with our DSMB, determined that they should not be used to define intervention safety or our modified intention to treat dataset.*

10. L. 298: provide reference for the SD value for haemoglobin concentration (or justify otherwise). Does this SD refer to each group (assuming equal group variance), or to the difference in haemoglobin concentration between groups? If it refers to SD within each group, a value of 17 g/L seems high considering that they will be analysed by Sysmex haematology analyser (L. 432).

Response: *We have added references for the SD value (Lines 340 to 342), which was based on HemoCue measurements at both sites. We first considered each site's SD separately and then selected the most conservative (highest) estimate across both sites for the sample size calculation, assuming equal variance across treatment arms. As a higher SD requires a larger sample size, this is a conservative assumption. If true variability is lower when measured by CBC, this will result in greater power.*

11. L. 299: provide clarify if, as I suspect, the sample size of 1,600 is based on the total number (i.e. both intervention groups) within each country (hence, 3,200 in the two countries combined).

    Response: *We have clarified that this is 1,600 women per country, i.e., 3,200 women in total.*

12. L. 317-318, 368-373: at baseline and during visits in the windows of 20<24 and 30<34 weeks, Burkinabe women will be tested for malaria using rapid dipstick test. Will positive cases be treated using artemisinin-based combination therapy (2nd and 3rd trimester) or quinine-clindamicin (1st trimester), in accordance with WHO guidelines? Will women also receive intermittent preventive treatment with sulphadoxine-pyrimethamine through their pregnancy?

    Response: *We have provided further information on malaria treatment, including intermittent preventive treatment, in the "Study Visits" section of the manuscript (Lines 363 to 371 and Lines 424 to 326).*

13. L. 438: Pakistani women will also be tested for malaria, but it is not clear from the description of the ANC visits at what time points.

    Response: *We have added the following in Lines 511 to 513: Both haemoglobinopathy and malaria testing will be done at baseline only, to inform ancillary analyses on the aetiology of anaemia in this setting. Results of these tests will be provided to women, who will be referred for counselling or treatment, respectively.*

14. L. 390-391: indicate whether the ferritin assay is qualitative, quantitative or semi-quantitative.

    Response: *The point-of-care ferritin is a quantitative assay. We have expanded our description in Lines 460 to 462 to say: "A capillary blood sample will be taken by heel prick from the infant and tested on site for Hb using the HemoCue Hb 301, ferritin using a*

quantitative immunochromographic test run on the Easy Reader+ platform (Veda-Lab, Alençon, France).

15. L. 424-425, 437-438: please describe ethical issues around the testing for haemoglobinopathies (e.g., sickle cell status) and how they will be dealt with. In particular, will participants always be informed about the results?

    Response: *We have provided further details on the haemoglobinopathy testing and how these results will be handled in the "Biospecimen Analysis" section of the manuscript (Lines 521 to 528).*

16. L. 448-454 (see also my first comment): when will you consider non-inferiority? PP often considered primary or at least co-primary for inference; non-inferiority is typically concluded only if PP analysis shows non-inferiority and ITT analysis is consistent (does not contradict PP).

    Response: *We agree that, for many non-inferiority trials, PP analysis provides a more conservative assessment. However, as differential adherence between trial arms is possible in our trial, PP analysis may introduce bias resulting from differential post-randomization exclusions. Therefore, while the results of both analyses will be examined for consistency, the ITT analysis will be considered primary. We have expanded the statistical analysis section to include this information, which is also available in our Statistical Analysis Plan.*

17. Statistical analysis: The protocol proposes pooling treatment effects across the two countries. This is acceptable if an overall treatment effect is the target estimand, but this should be explicitly stated and justified. The authors should clarify whether country is considered a potential effect modifier and specify how between-country heterogeneity will be assessed (e.g., inclusion of a treatment-by-country interaction term and reporting of country-specific estimates). With only two countries, random-effects pooling should be used cautiously and, if applied, treated as exploratory or sensitivity analysis rather than primary inference. It would seem to make more sense to handle country as a fixed effect.

    Response: *Country-specific difference in mean Hb concentration (co-administration minus separate administration), estimated using simple linear regression within each country, with country-specific effects combined as a pooled average treatment effect using both fixed-effects and random-effects inverse variance approaches (DerSimonian-Laird). Because only two countries contribute to the pooled analysis, we cannot precisely estimate between-country heterogeneity. We will therefore report pooled estimates under both fixed-effect and random-effects assumptions. Country-specific results are the primary*

basis for the non-inferiority decision; the pooled estimate is a secondary summary. The fixed-effect model provides an estimate under the assumption of a common treatment effect across countries, while the random-effects model incorporates a heterogeneity term, though we acknowledge that with only two countries this should be interpreted cautiously. Together, these secondary summaries will present the range of plausible pooled effects without relying on an imprecise estimate of between-country heterogeneity or assuming a common effect across countries. We have clarified this in Lines 558 to 568 of the "Statistical methods" section. We have also specified this in the Estimands framework that was added based on the reviewer's feedback (Supplemental Table 1).

18. L. 486-88: A log-binomial regression model with a log link estimates risk ratios, not risk differences. I also presume that 'risk', at least in some cases (e.g., anaemia, iron deficiency), in fact refers to 'prevalence'?

    Response: *Yes, in most cases we are referring to prevalence. We have modified the estimated "risk differences" to "prevalence ratios." We will retain this for consistency even where we have incident binary outcomes.*

19. References 12, 13: issue/page numbers are missing.

    Response: *Thank you for identifying these. We have updated the reference list.*
